# Does growth path influence beef lipid deposition and fatty acid composition?

**DOI:** 10.1371/journal.pone.0193875

**Published:** 2018-04-03

**Authors:** Ana S. H. Costa, Paulo Costa, Susana P. Alves, Cristina M. Alfaia, José A. M. Prates, Veronica Vleck, Isabelle Cassar-Malek, Jean-François Hocquette, Rui J. B. Bessa

**Affiliations:** 1 CIISA – Centro de Investigação Interdisciplinar em Sanidade Animal, Faculdade de Medicina Veterinária, Universidade de Lisboa, Avenida da Universidade Técnica, Pólo Universitário do Alto da Ajuda, Lisboa, Portugal; 2 CIPER – Faculdade de Motricidade Humana, Universidade de Lisboa, Estrada da costa, Cruz Quebrada-Dafundo, Lisboa, Portugal; 3 INRA, UR 1213, Unité de Recherches sur les Herbivores (URH), Theix, Saint-Genés Champanelle, France; 4 Clermont Université, VetAgro Sup, UMR1213, Herbivores, Clermont-Ferrand, France; University of Illinois, UNITED STATES

## Abstract

Despite the recent advances in transcriptomics, gene expression studies addressing cattle´s skeletal muscle adaptations in response to compensatory growth are warranted, particularly regarding lipid metabolism due to its impact in meat sensory and nutritional traits. In the present study, in comparison to *ad libitum* feeding, a period of feed restriction was used in order to understand the changes in bull´s lipid metabolism and gene expression of the adipogenic and lipogenic pathways after re-alimentation. Thus, 40 young Alentejana bulls were either fed *ad libitum* (CG group) from 9 to 18 months of age or subjected to food restriction from 9 to 15 months of age, and fed *ad libitum* until 24 months of age (DG group). The intramuscular fat (IMF) and total fatty acids (FA) contents were similar between groups. The major FA (>2%) contents were similar (16:0, 16:1*c*9, 18:1*c*9 and 18:2n-6) between treatments with the exception of 18:0 content that was 15% lower in DG than in CG and 20:4n-6 that tended to be greater on DG bulls. Regarding minor FA (<2%), the DG group presented greater proportions (*P*<0.01) of 17:1*c*9, 18:1*t*9, 18:1*t*10 (, 18:1*c*11), 18:1*c*13, 18:3n-6, 22:0, 22:4n-6 and 22:6n-3 and lower (*P*<0.05) proportions of 20:0, 18:1*t*16+*c14*, and branched chain FA (iso-15:0, anteiso-15:0, iso-16:0 and anteiso-17:0) than the CG group. Delta-9 desaturase activity indices were consistently greater (*P*<0.05) in DG, when compared to the CG group. Regarding microarray analysis, differentially expressed genes between CG and DG bulls were grouped in 5 main biological functions: lipid and nucleic acid metabolisms, small molecule biochemistry, molecular transport and translational modification. Discontinuous growth down-regulated the expression of *ACACB* (FC (fold-change) = 1.32), *FABP3* (FC = 1.45), *HADHA* (FC = 1.41) and *SLC37A4* (FC = 1.40) genes, when compared to the CG system (FDR<0.05). In contrast, in the CG bulls, the expression of *ELOVL5* (FC = 1.58) and *FASN* (FC = 1.71) was down-regulated when compared to DG bulls. These results were confirmed to be significant (*P*<0.05) in the case of *ELOVL5*, *FASN* and *SLC37A4*, and almost significant for *FABP3* by qRT-PCR analysis. The *SCD1* and *SCD5* gene expressions were not found to be affected by growth path. These results contribute to the still scarce knowledge about the mechanisms involved in fatty acid metabolism during compensatory growth which have decisive role on meat quality produced in Mediterranean areas.

## Introduction

Compensatory growth is a physiological process whereby growth accelerates after a period of restricted development, usually due to reduced feed intake [1]. The compensatory growth has been used for a long time in traditional cattle production in Mediterranean areas, taking advantage of the local natural conditions and favoring a sustainable agriculture and maintaining biodiversity in rural areas. Additionally, the discontinuous growth (DG) system presents economic benefits, given that pasture feeding is less expensive than grain and that consumers prefer beef from animals raised on pasture [2]. Moreover, when included in a management strategy, it may be possible to obtain some meat quality benefits because compensatory growth has been shown to influence meat yield and tenderness [3].

The factors contributing to compensation in cattle include: increased feed intake, increased gut-fill weight, and/or enhanced feed efficiency. The response varies with the length of the undernutrition and re-feeding periods and the animal’s development stage, being better expressed when feed restriction occurs at a relatively late growth-stage [1,4,5].

During compensatory growth, the animals experience several physiological and metabolic changes with impact on muscle characteristics and metabolic enzyme capacity [6]. It has been demonstrated that nutritional restriction has a significant effect on protein turnover and cytoskeletal metabolism [7]. Lehnert et al. [8] showed that severe undernutrition down-regulates genes coding for muscle structural proteins, extracellular matrix and metabolic enzymes, particularly those associated with the glycolytic pathway. In recent years, the physiological adaptations following feed restriction have been studied at a molecular level, making use of the advances in transcriptomics [8–10]. However, gene expression experiments addressing cattle´s skeletal muscle adaptations in response to compensatory growth are still scarce and studies on the genetic and metabolic adaptations underlying compensatory growth are needed to identify adipogenic and lipogenic genetic pathways related to intramuscular fat deposition [11] and muscle fatty acid composition [12].

Previous results from this experiment showed that growth path had no significant effect on intramuscular fat (IMF) content across eight major muscles, including *longissimus thoracis* (Lt) muscle [13]. This figure suggests that the re-feeding period was sufficient to neutralize the effects of feed restriction on muscle IMF content but raises the question of which genes are involved in this process. Thus, the present investigation aims to identify differentially expressed genes and their role in Lt muscle with similar IMF content from autochthonous bulls raised according to distinct growth paths. A greater understanding of the molecular control regulating FA deposition in CG animals could provide valuable scientific information with potential applicability in breeding programs focused in the improvement of beef quality (reviewed by Cassar-Malek et al. [14]) and contribute to the surviving of low growth rate breeds produced according to traditional systems, such as Alentejana. This autochthonous breed can be found in the Southern of Portugal and is mainly used for meat production. In the past, it was widely used as tractive force in the vast plains of Alentejo. Alentejana breed has genealogical and phenotypic similarities with the Spanish Retinta and the Portuguese Mertolenga breeds. To investigate the effects of the production system on muscle fatty acid composition and related gene expression, 40 young Alentejana bulls were either fed *ad libitum* (CG group) from 9 to 18 months of age or subject to food restriction from 9 to 15 months of age, and fed *ad libitum* until 24 months of age (DG group).

## Materials and methods

### Animals and experimental design

Animals were handled in accordance to Faculdade de Medicina Veterinária-Universidade de Lisboa (FMV-UL) and national guidelines covering animal experiments, and approved by the Animal Care Committee of the National Veterinary Authority (Direcão-Geral de Veterinária) following the appropriate European Union guidelines (Directive 86/609/EEC).

The experiment is described in detail in Costa el al. [13]. Briefly, forty pure bred 9.0±0.46 (mean ± S.D.) month-old Alentejana male calves with 239±45 kg (mean ± S.D.) of live weight were randomly allocated into four adjacent pens (two pens per experimental group) and submitted to two distinct feeding regimes. In the continuous growth production system (CG), animals were fed *ad-libitum* with concentrates plus hay throughout the trial and slaughtered at 18 months of age. In the discontinuous growth production system (DG), animals were fed *ad-libitum* on hay until 15 months of age, following the same diet provided to the CG group (concentrates plus hay) from 15 to 24 months of age. The pens and the feed bunks were large in order to reduce animal´s interaction and limitation to feed and to simulate the traditional systems were the young bulls graze freely. The animals were individually weighted every 14 days during the experiment and slaughtered when they reached 18 and 24 months of age for CG and DG groups, respectively. Six animals from DG group were discarded from the trial due to digestive disorders that impaired growth related to diet transition.

### Slaughter and sampling

After stunning with captive bolt, animals were slaughter by exsanguination and the carcasses were dressed. Afterwards, samples of *longissimus thoracis* muscle (Lt) were collected from the right side of carcass for gene expression analysis at the 12^th^ thoracic vertebra level, rinsed with sterile RNAse-free cold-water solution, cut into small pieces (thickness of ~0.3 cm), snap-frozen in liquid nitrogen and subsequently stored at -80 °C. Longissimus muscle was chosen due to its high economic value and because it is the most widely studied muscle in meat research and hence, comparison of results obtained across different studies.

After an ageing period of 7 days, carcasses were jointed and two 2.5 cm thick steaks were taken from the space between de 10^th^ and 12^th^ dorsal vertebrae in the Lt muscle, trimmed of visible adipose and connective tissues and minced. About 100 g were vacuum packed and frozen at -30°C for subsequent determination of intramuscular fat content and fatty acid analysis.

### Lipid extraction and methylation of fatty acids

Meat samples were lyophilized (- 60 °C and 2.0 hPa) to constant weight, using a lyophilizator (Edwards High Vacuum International, West Sussex, UK), kept dry at room temperature and analysed within two weeks. For determination of intramuscular lipid content (equivalent to intramuscular fat (IMF) content), total lipids were extracted from lyophilized meat samples (*ca*. 250 mg) as described by Carstens et al. [5]. Total lipids were measured gravimetrically, in duplicate, by weighing the fatty residue obtained after solvent evaporation.

### Fatty acid analysis

Fatty acids were transesterified with sodium methoxide followed by hydrochloric acid in methanol (1:1 vol/ vol). Fatty acid methyl esters (FAME) were extracted twice with 3 mL of hexane and pooled extracts were evaporated, under a stream of nitrogen at 35 °C, until a final volume of 2 mL. The resulting FAME were then analyzed by gas chromatography using a fused-silica capillary column (CP-Sil 88; 100 m × 0.25 mm i.d. × 0.20 μm film thickness; Chrompack, Varian Inc., Walnut Creek, CA, USA), equipped with a flame-ionization detector, as described by [16]. Quantification of FAME used nonadecanoic acid (19:0) as internal standard, which was added to the lipids prior to saponification and methylation. Fatty acid composition was expressed as g/100 g of total fatty acid content, assuming a direct relationship between peak area and fatty acid methyl ester weight.

### Hybridisation studies

RNA extractions were performed using RNeasy Lipid Tissue Mini Kit (Qiagen, Hilden, Germany) according to the manufacturer’s protocol. After extraction, total RNA was quantified with a Nanodrop ND.1000 spectrophotometer (ThermoScientific, World Headquarters Location, Waltham, USA). RNA integrity was evaluated with the 2100 bioanalyzer (Agilent Technologies, Massy, France) and the RNA 600 LabChip kit. The total RNA was amplified and labelled with Cyanine 3 using Agilent’s Low RNA Input Linear Amplification Kit, PLUS, One-Color (Agilent Technologies) following the detailed protocol described by Agilent.

Hybridisation studies were performed as previously described by Hocquette et al. [18] and Hiller et al. [17]. Briefly, 500 ng of total RNA was reverse transcribed to double-strand cDNA using a poly dT-T7 promoter primer. cDNA products were then used as templates for in vitro transcription to generate fluorescent cRNA. Labelled cDNAs were finally purified using QIAGEN’s RNeasy mini spin columns and eluted in 30 μl of nuclease-free water. After amplification and labelling, cDNA quantity and cyanine incorporation were determined using a Nanodrop ND.1000 spectrophotometer (ThermoScientific). For each hybridisation, 600 ng of Cyanine 3 labelled cRNA were fragmented and hybridised at 65°C for 17 hours to an Agilent 8 x 15K custom Oligo Microarray. After washing, microarrays were scanned using an Agilent DNA G2505B scanner. Feature extraction 9.1 software (Agilent Technologies) was used to assess fluorescent hybridisation signals.

The total number of probes was 10257, of which 1614 were control probes used for normalisation. Data extraction was performed using the Feature Extraction (Agilent Technologies). Generally, probes for the same gene gave similar results. For each array, normalisation was applied with the median of the 1614 control probes whose average expression level did not significantly differ between animal groups. Normalisation was then calculated per probe from the median of the probe obtained from all arrays. This ensured to use the same scale for all probes. Finally, a log2 transformation was done.

### Pathway analysis

Microarray gene expression profile analysis and biological pathways that were significantly over-represented among differentially expressed genes were identified using GEPAS (Gene Expression Profile Analysis Suite (http://bioinfo.cipf.es/)). The differentially expressed genes functional annotation was analysed using the online tool Panther (http://www.pantherdb.org/). The set of differentially expressed genes was then applied to test KEGG pathways (http://www.genome.jp/kegg/pathway.html) for over- or under-representation. The significant KEGG pathway maps were examined for significant differentially expressed genes. The molecular functions and biochemical pathways were further analysed using Ingenuity pathway analysis 7.0 (IPA, Ingenuity Systems Inc., Redwood City, CA; http://www.ingenuity.com/), a web-based software application that enables identification of over-represented biological mechanisms, pathways and functions most relevant to experimental datasets or genes of interest.

### Gene expression analysis using the real-time reverse transcription quantitative PCR (RT-qPCR)

For microarray validation purposes, RT-qPCR was used to measure transcript abundance of genes that were identified as either statistically significantly differentially expressed or as genes of interest.

Total RNA was extracted from Lt muscle samples using Trizol reagent (Invitrogen, Carlsbad, CA, USA) and purified with the RNeasy Mini Kit (Qiagen, Hilden, Germany), according to the manufacturer’s protocol. An additional step of DNase digestion was included, using the RNase-free DNase Set (Qiagen, Hilden, Germany). A NanoDrop Spectrophotometer (ND-2000c, Willmington, DE, USA) was used to analyze RNA samples for quantity (OD260 nm) and purity (OD260nm/OD280nm). The RNA aliquots were stored at –80 °C until further analysis.

All RNA samples were reverse transcribed to cDNA using the High Capacity cDNA Reverse Transcription Kit (Applied Biosystems, Foster City, CA, USA) following the manufacturer’s protocol. Each 20 μl RT reaction contained 800 ng of RNA template, 50 nM random RT Primer, 1×RT buffer, 0.25 mM of each dNTPs, 3.33 U/μl multiscribe reverse transcriptase and 0.25 U/μl RNase inhibitor, at temperatures of 25 °C for 10 min, 37 °C for 120 min, and 85 °C for 5 sec. cDNA aliquots were diluted 1:20 and stored at –20 °C.

The RT-qPCR of eight genes of interest was performed with the StepOne Plus^™^ Real-Time PCR System, using the Power SYBR^®^ Green master mix (both Applied Biosystems, Foster City, CA, USA). Gene expression levels were detected for target genes depicted in Table 1, while TATA box binding protein (TBP) was selected as an endogenous control from a set of candidate housekeeping genes. Expression stability of candidate housekeeping genes was evaluated using geNorm and NormFinder, following the procedures described in [19] and [20], respectively.

**Table 1:** Specifications of oligonucleotides used for RT-qPCR analysis.

Gene symbol	Acc. Number	Primer Forward	Primer Reverse	Amplicon length (bp)	Tm (Fw/Rv)
ACACB	NM_001205333.1	ACTCCGCCCTCAAGACCATC	GGCTGATGTGCTGGCTGAC	90	60.0/58.9
ELOVL5	NM_001046597.1	CCCTCTCGGTTGGTTGTATTTC	GTGGTCCTTTTGGTGCTCTCTC	127	58.5/58.8
HADHA	NM_174335.2	CCACAGAGGGAGACATCGGT	ACGGGGTGAACTGTTTTCCA	150	58.5/58.9
FABP3	NM_174313.2	GAGACCACAGCAGATGACAGGA	CCATTTCCCGCACAAGTGAT	112	58.9/59.4
FASN	NM_001012669.1	ATGGCGTTCCACTCCTACTTCA	CTCTCCTGCCACTGGGTCTC	137	59.6/58.4
SCD1	NM_173959.4	CCATCAACCCCCGAGAGAAT	AAGGTGTGGTGGTAGTTGTGGAA	143	59.7/59.3
SCD5	NM_001076945.1	CGGGCAACCAAGCAGATG	GGGCGCAGATGGAGGAT	83	59.8/58.1
SLC37A4	XM_001253040.4	CATCTCACCCCATCCTCACC	GGACAATCAGGGTGGGGC	120	58.7/59.7
*Housekeeping*					
TBP	NM_001075742.1	GTTCTGGGAAGATGGTGTGCA	GTTCTGGGAAGATGGTGTGCA	73	59.5/58.3

ACACB: acetyl-CoA carboxylase beta; ELOVL5: fatty acid elongase 5; HADHA: hydroxyacyl-CoA dehydrogenase/3-ketoacyl-CoA thiolase/enoyl-CoA hydratase, alpha subunit; FABP3: fatty acid binding protein 3; FASN: fatty acid synthase; SCD1: stearoyl-CoA desaturase 9; SCD5: stearoyl-CoA desaturase 5; SLC37A4: solute carrier family 37, member 4; TBP: TATA box binding protein

Primer sequences (Table 1) were designed to fall across exon–exon junctions with Primer3 (version 0.4.0; Rozen and Skaletsky, 2000) and Primer Express 3.0 software (Applied Biosystems, Foster City, CA, USA). Homology alignments for the selected primers against publicly available databases using BLASTN at the National Center of Biotechnology Information showed that these primers matched only the sequence to which they were designed. Dissociation curve analysis was performed to rule out amplification of non-specific products. Each PCR reaction was performed, in duplicate, in a final volume of 12.5 μl with 1 μl forward and reverse primers (160 nM), 6.25 μl *Power* SYBR Green PCR Master Mix (2×) (Applied Biosystems, Foster City, CA, USA), 1 μl diluted cDNA (1:10) and nuclease-free water up to volume.

Real-time PCR amplification efficiency was examined by generating a standard curve from 5-fold serial dilutions of pooled cDNA. The relative expression (RE) levels were calculated as a variation of the Livak method [21], corrected for variation in amplification efficiency (E = 10^−1/*slope*^), as shown in Eq 1.

RE = Eendogenous(CT,endogenous)Etarget(CT,target) (1)

### Western blot analysis of SCD

Using a mechanical disruption method, 80 mg of bovine muscle or mouse liver (control) were lysed and homogenized by high-speed agitation in the TissueLyser in the presence of glass beads and ice-cold lysis buffer. Lysis buffer consisted of freshly prepared (10 nM Tris-HCl, pH 7.6; 5mM MgCl_2_; 1.5 mM Kac). Just prior to use, 2 mM dithiothreitol (Promega Corporation, Madison, WI, USA) and one tablet of protein inhibitor cocktail (Complete; Roche Applied Science, Mannheim, Germany), were added per 10 ml of buffer. To the aliquot obtained after tissue homogenization an equal volume of 10 mM Tris (pH 7.6) with 1% NP-40 (Calbiochem, San Diego, CA, USA) was added, incubated on ice and sonicated. After centrifugation at 3200 g for 10 min, the supernatant was saved for protein separation. Protein quantification was performed by the BioRad protein assay (BioRad, Ref. 500–0006, Hercules, CA, USA). Total proteins were loaded on a 12% polyacrylamide gel electrophoresis for SCD detection. Membranes were processed for protein detection using Super Signal substrate (Pierce, Rockford, IL, USA).

Four different commercial antibodies against murine SCD, and cross-reactivity with the bovine species, were tested (Santa Cruz Ref. sc-30081, Sigma Ref. AV32797, Cell Signaling Ref. 2438, and Abcam Ref. ab39969) following the manufacturers’ guidelines. Despite being able to detect SCD in the mouse liver samples, none of the antibodies detected bovine SCD in the samples tested.

### Statistical analysis

For statistical analyses, the PROC MIXED of SAS software version 9.4 (SAS Institute Inc., 2009) was used. The IMF and fatty acid data were analysed with a statistical model that included the growth path type as single fixed effect. The homogeneity of variances between experimental groups were tested for heteroscedasticity and when needed the two variances were accommodated in the model using the GROUP option of the REPEATED statement from PROC MIXED. Results were expressed as least square least square mean ± standard error of the mean (SEM). The gene expression data was expressed as fold change and analysed using the PROC MIXED of SAS software. The false discovery rate (FDR) was calculated according to McLachlan et al. [54]. Differences were declared significant at *P*<0.05 and tendencies discussed at *P*<0.10.

## Results

Differences in live weight gain, feed intake, and animal performance are described in detail by Costa et al. [13, 22]. Briefly, the average daily gain (ADG) from 9 to 15 months of age was 1678 g/day and 99 g/day in CG and DG bulls, respectively. The ADG in CG animals is within the values reported for Alentejana breed [55]. In the period from 15 to 18 months of age, the ADG was 1204 g/day and 1691 g/day in CG and DG bulls, respectively. Animals subjected to the restricted feeding regime (DG) had 2 additional growth periods wherein the ADG was around 1315 and 1082 kg/day from 18 to 21 months of age and from 21 to 24 months of age, respectively. On average, the CG bulls were 37 kg heavier than DG animals (643 kg vs 606 kg, P<0.08) whereas body composition did not differ between the two groups of animals [13].

### Intramuscular fat and fatty acid composition

The IMF, fatty acid (FA) contents and composition of Lt muscle are depicted in Table 2. The IMF and total FA contents were similar between experimental groups (*P*>0.40). Proportions of major FAs (i.e. > 2% of total FA) were similar (*P*>0.05) (16:0, 16:1*c*9, 18:1*c*9 and 18:2n-6) between treatments with the exception of 18:0 which had a 15% lower proportion (P<0.001) in DG than CG and 20:4n-6 proportion that tended (P = 0.057) to be higher in DG than in CG group. The DG bulls had higher proportions of 17:1*c*9, 18:1*t*9, 18:1*t*10, 18:1*c*11, 18:1*c*13, 18:3n-6, 22:0, 22:4n-6 and 22:6n-3 and lower proportions of 20:0, 18:1*t*16, and branched chain FA (iso-15:0, anteiso-15:0, iso-16:0 and anteiso-17:0) than the CG group. From all FA, the major difference was observed for a rumen biohydrogenation intermediate, the 18:1*t*10, with a proportion 3.7 times higher in DG than in CG meat samples. Variance of 18:1*t*10 was also significantly larger (*P* < 0.0001) in DG than in CG (0.086 vs. 2.018) (data not shown). The ratio between 18:1*t*10 and 18:1*t*11 was larger (*P* < 0.004) in DG (5.85 ± 1.44) than in CG (1.01 ± 0.153) group. The variance of the 18:1t10/18:1t11 ratio was 100 times (*P*<0.0001) larger in DG (35.1) than in CG (0.35) group. The large and significant differences observed for 18:0 and 18:1*t*10 proportions are directly reflected in the partial sums of total saturated FA (SFA) and trans-FA proportions, respectively.

**Table 2:** Intramuscular fat (IMF, g/100g meat), total fatty acids (total FA, g/100g muscle) and fatty acid composition (% of total FA) of muscle in continuous growth (CG) and discontinuous growth (DG).

	Group			*P-value*
	CG (n = 20)	DG (n = 16)		
IMF	1.87±0.15	1.93±0.17		0.807
Total FA	1.46±0.12	1.60±0.13		0.437
*Fatty acids*				
14:0	2.15±0.11	2.06±0.10		0.608
14:1c9	0.32±0.03	0.36±0.03		0.404
*i*-15:0	0.09±0.005	0.06±0.004		<0.001
*a*-15:0	0.15±0.006	0.12±0.005		0.009
15:0	0.29±0.01	0.32±0.01		0.183
*i*-16:0	0.13±0.007	0.11±0.007		0.017
16:0	24.1±0.4	23.6±0.3		0.362
16:1c7	0.17±0.003	0.18±0.006		0.237
16:1c9	2.57±0.13	2.76±0.15		0.376
*i*-17:0	0.35±0.03	0.31±0.02		0.200
*a*-17:0	0.51±0.03	0.43±0.02		0.033
17:0	0.96±0.03	1.01±0.03		0.266
17:1c9	0.60±0.02	0.79±0.03		<0.001
*i*-18:0	0.10±0.004	0.09±0.003		0.187
18:0	17.9±0.4	15.3±0.4		<0.001
18:1t6+t8	0.16±0.01	0.19±0.01		0.052
18:1t9	0.20±0.01	0.26±0.02		0.010
18:1t10	0.52±0.07	1.91±0.34		<0.001
18:1t11	0.53±0.03	0.40±0.02		0.003
18:1c9	29.6±0.8	29.3±0.8		0.731
18:1c11	1.68±0.06	1.93±0.07		0.003
18:1c12	0.33±0.04	0.30±0.04		0.658
18:1c13	0.14±0.009	0.19±0.01		0.004
18:1t16+c14	0.13±0.004	0.09±0.006		<0.001
18:1c15	0.04±0.04	0.05±0.04		0.087
18:2n-6	9.99±0.59	9.95±0.55		0.967
18:3n-3	0.43±0.02	0.41±0.02		0.471
18:3n-6	0.05±0.005	0.08±0.006		<0.001
20:0	0.11±0.003	0.09±0.002		0.005
20:1c11	0.04±0.002	0.04±0.002		0.984
CLA(c9t11)	0.09±0.008	0.10±0.009		0.722
20:2n-6	0.08±0.004	0.08±0.004		0.169
20:3n-9	0.11±0.01	0.11±0.009	0.01	0.566
20:3n-3	0.02±0.001	0.03±0.002	0.002	0.157
20:4n-6	2.75±0.19	3.35±0.25	0.22	0.057
20:5n-3	0.13±0.02	0.14±0.02	0.02	0.491
22:0	0.48±0.03	0.66±0.05		0.003
22:4n-6	0.31±0.02	0.38±0.03	0.02	0.029
22:5n-3	0.35±0.03	0.41±0.03	0.03	0.198
22:6n-3	0.03±0.004	0.05±0.004	0.004	0.026
*Partial sums*				
SFA	46.0±0.7	43.1±0.6	0.6	0.002
*cis*MUFA	35.5±0.8	35.9±0.8	0.8	0.765
TFA	1.64±0.10	2.95±0.33		0.001
BCFA	1.34±0.07	1.13±0.05	0.06	0.013
PUFA	14.2±0.8	15.0±0.8	0.8	0.529
n-3	0.53±0.05	0.62±0.05	0.05	0.199
n-6	13.2±0.8	13.9±0.8	0.8	0.550
*Desaturation indices*				
ID14	12.7±0.7	14.6±0.8	0.8	0.086
ID16	9.39±0.30	10.7±0.38	0.36	0.016
ID17	38.1±0.1	44.3±0.2	0.1	<0.001
ID18	61.6±0.7	66.0±0.9	0.8	<0.001
IDCLA	15.1±1.1	20.1±1.6	1.4	0.017

IMF = intramuscular fat; FA = fatty acids; SFA = saturated fatty acids (sum of 14:0, 15:0, 16:0, 17:0, 18:0 and 20:0); cisMUFA = monounsaturated fatty acids (sum of 14:1c9, 16:1c7, 16:1c9, 17:1c9, 18:1c9, 18:1c11, 18:1c12, 18:1c13, 18:1c15, 19:1 and 20:1c11); TFA = trans fatty acids (sum of 18:1t6-t8, 18:1t9, 18:1t10, 18:1t11, 18:1t12, 18:1t16, c14 and 18:2t11c15); BCFA = branched chain fatty acids (sum of i-14:0, i-15:0, a-15:0, i-16:0, i-17:0, a-17:0 and i-18:0 (*a*- = anteiso *i*- = iso)); PUFA = polyunsaturated fatty acids (sum of 18:2n-6, 18:3n-6, 18:3n-3, CLA, 20:3n-3, 20:5n-3, 22:5n-3, 22:6n-3; 20:2n-6, 20:4n-6 and 22:4n-6); n-3 = sum of 18:3n-3, 20:3n-3, 20:5n-3, 22:5n-3 and 22:6n-3; n-6 = sum of 18:2n-6, 18:3n-6, 20:2n-6, 20:4n-6 and 22:4n-6; ID14:0 = (14:1c9×100)/(14:0+14:1c9); ID16:0 = (16:1c9×100)/(16:0+16:1c9); ID18:0 = (18:1c9×100)/(18:0+18:1c9); IDCLA = (18:2c9,t11×100)/(18:1t11+18:2c9,t11). Means in the same row with different superscripts are statistically different (P<0.05);

The delta-9 desaturase activity indices, computed by using FA ratios relating each pair of product and substrate, were consistently higher in the DG, when compared to the CG group, except the ID14 that tended to be higher (P = 0.086).

### Differential analysis on microarray

Among the genes found to be expressed in Lt muscle, 87 transcripts were differentially expressed between restricted and control group. Full details of gene name, function, accession number, fold-change and *P-value* for all differentially expressed transcripts are listed in S1 and S2 Tables.

### Functional annotation

Among the genes analysed by microarrays that were detected to be differentially expressed between DG and CG group, 87 were submitted to annotation analyses. When compiling the lists of genes expressed differentially between experimental groups, the software Ingenuity Analysis Pathways 7.0 (IPA, Ingenuity System^®^, http://www.ingenuity.com) highlighted several biological functions (Table 3) that can be grouped in 5 main biological functions: lipid and nucleic acid metabolisms, small molecule biochemistry, molecular transport and translational modification. The genes associated with lipid and nucleic acid metabolisms and small molecule biochemistry represented about 22% of the 87 genes considered for annotation analysis. A focus was made on genes involved in lipid metabolism to relate gene expression data to fatty acid composition of muscles. According to Gene Ontology, the most relevant biological functions associated with lipid metabolism are depicted in Table 4.

**Table 3:** Relevant biological functions identified from the annotation analysis.

Category	P-value	Molecules
Lipid Metabolism	1,52E-05-1,31E-02	*ACACB*, ***ACLY***, ***ANXA5***, *ATP1A2*, *CAT*, ***CAV2***, *CD36*, *COL18A1*, ***DLK1***, ***EEF1A1***, ELOVL5*, *ERCC1*, FABP3*, FASN*, *GNPAT*, HADHA*, *HIF1A*, HMGCR*, ***IGF2***, *KCNMA1*, LDLR*, PLAT*, *RAC1*, *SDHB*, *SLC37A4*, ***VIM***
Nucleic Acid Metabolism	1,52E-05-6,63E-03	*ACACB*, ***ACLY***, *CAT*, *DES*, *ERCC1*, FASN*, HMGCR*, HADHA*, ***PRDX5***
Small Molecule Biochemistry	1,52E-05-1,31E-02	*ACACB*, ***ACLY***, ***ANXA2***, ***ANXA5***, *ATF4*, *ATP1A2*, *CAT*, ***CAV2***, *CD36*, ***COL18A1***, *DES*, ***DLK1***, ***EEF1A1***, ELOVL5*, *ERCC1*, FABP3*, *GNPAT*, *GPX3*, HADHA*, *HIF1A*, HMGCR*, ***IGF2***, *KCNMA1*, LDLR*, ***LAMA4***, *PRDX5*, ***PLAT***, *RAC1*, *SLC37A4*, *SDHB*, ***VIM***
Molecular Transport	1,92E-05-9,17E-03	*ACACB*, ***ACLY***, *ALDOA*, ***ANXA5***, *ATF4*, *CAT*, *CD36*, ***COL18A1*, *DLK1***, ***EEF1A1***, ELOVL5*, *ERCC1*, FASN*, *FABP3*, HADHA*, *HIF1A*, HMGCR*, ***IGF2***, *KCNMA1*, LDLR*, ***LAMA4***, PLAT*, *PRDX5*, *RAC1*, *SDHB*, *SLC37A4*
Post-Translational Modification	3,52E-05-6,63E-03	***ANXA2***, *CAT*, GPX3*, HMGCR*, *KCNMA1*, *RPS19*

The biological interpretation of expression data was performed using Ingenuity Pathway Analysis 7.0 (IPA, Ingenuity Systems Inc., Redwood City, CA). The genes included in the analysis were shown to be differential between continuous growth (CG) and discontinuous growth (DG) by microarray. Genes are presented in alphabetical order for each category. The genes over expressed in Longissimus thoracis muscle from DG animals are in bold. Genes marked with asterisk (*) are among the top 10 with higher fold-change.

**Table 4:** Relevant biological functions associated with lipid metabolism identified from the annotation analysis using microarrays results.

Functions Annotation	*P-value*	Molecules
Accumulation of C18:2 fatty acid	2,51E-06	*CD36*, *ELOVL5*, *HADHA*
Concentration of lipid	3,64E-06	*ACACB*, *ACLY*, *ANXA5*, *CD36*, *COL18A1*, *DLK1*, *EEF1A1*, *ELOVL5*, *ERCC1*, *FABP3*, *FASN*, *HADHA*, *HIF1A*, *HMGCR*, *IGF2*, *KCNMA1*, *LDLR*, *PLAT*, *RAC1*, *SLC37A4*
Modification of palmitic acid	6,73E-06	*ACACB*, *CD36*, *FABP3*, *FASN*, *HIF1A*
Conversion of acyl-coenzyme A	2,94E-05	*ACLY*, *FASN*, *HMGCR*
Uptake of 15-(4-iodophenyl)-3-methylpentadecanoic acid	4,05E-05	*CD36*, *FABP3*
Concentration of acylglycerol	5,89E-05	*ACACB*, *ACLY*, *CD36*, *COL18A1*, *DLK1*, *ELOVL5*, *ERCC1*, *FASN*, *IGF2*, *LDLR*, *SLC37A4*
Accumulation of fatty acid	6,26E-05	*CD36*, *ELOVL5*, *HADHA*, *SDHB*
Homeostasis of lipid	1,06E-04	*ACACB*, *CD36*, *FABP3*, *GNPAT*, *HMGCR*, *LDLR*, *SLC37A4*
Accumulation of lipid	1,15E-04	*CD36*, *ELOVL5*, *ERCC1*, *FASN*, *HADHA*, *HMGCR*, *IGF2*, *LDLR*, *SDHB*
Oxidation of palmitic acid	1,20E-04	*ACACB*, *CD36*, *FABP3*, *HIF1A*
Accumulation of linoleic acid	1,21E-04	*CD36*, *ELOVL5*
Accumulation of medium chain dicarboxylic acid	1,21E-04	*HADHA*, *SDHB*
Synthesis of malonyl-coenzyme A	1,21E-04	*ACACB*,*FASN*
Synthesis of fatty acid	1,37E-04	*ACACB*, *ACLY*, *CAV2*, *ELOVL5*, *FASN*, *IGF2*, *LDLR*, *RAC1*, *VIM*
Metabolism of acylglycerol	1,40E-04	*CAT*, *CD36*, *FASN*, *IGF2*, *LDLR*, *SLC37A4*
Concentration of triacylglycerol	1,41E-04	*ACACB*, *ACLY*, *CD36*, *COL18A1*, *DLK1*, *ELOVL5*, *ERCC1*, *FASN*, *LDLR*, *SLC37A4*
Synthesis of acyl-coenzyme A	2,28E-04	*ACACB*, *ACLY*, *FASN*
Metabolism of triacylglycerol	2,33E-04	*CAT*, *CD36*, *FASN*, *LDLR*, *SLC37A4*
Fatty acid metabolism	2,97E-04	*ACACB*, *ACLY*, *CAV2*, *CD36*, *ELOVL5*, *FABP3*, *FASN*, *IGF2*, *LDLR*, *RAC1*, *SDHB*,*VIM*
Accumulation of palmitic acid	4,00E-04	*CD36*,*HADHA*
Concentration of fatty acid	5,51E-04	*ACACB*, *ACLY*, *CD36*, *DLK1*, *ELOVL5*, *FABP3*, *HADHA*, *IGF2*, *LDLR*
Steroid metabolism	7,79E-04	*CAT*,*HMGCR*,*IGF2*,*KCNMA1*,*LDLR*,*SLC37A4*,*VIM*
Accumulation of monounsaturated fatty acids	8,32E-04	*CD36*, *HADHA*
Metabolism of acetyl-coenzyme A	8,32E-04	*ACACB*, *ACLY*
Homeostasis of cholesterol	9,84E-04	*CD36*, *HMGCR*, *LDLR*, *SLC37A4*
Synthesis of lipid	1,29E-03	*ACACB*, *ACLY*, *CAV2*, *EEF1A1*, *ELOVL5*, *FABP3*, *FASN*, *GNPAT*, *HMGCR*, *IGF2*, *LDLR*, *RAC1*, *VIM*
Concentration of phosphatidic acid	1,41E-03	*ANXA5*, *CD36*, *EEF1A1*, *HIF1A*, *LDLR*
Deposition of lipid	1,43E-03	*FASN*, *HIF1A*, *LDLR*
Oxidation of lipid	1,43E-03	*ACACB*, *CAT*, *CD36*, *FABP3*, *HADHA*, *HIF1A*
Lipolysis	1,63E-03	*ACACB*,*CD36*, *COL18A1*, *EIF4EBP1*
Concentration of choline-phospholipid	1,81E-03	*ANXA5*, *CD36*, *LDLR*
Conversion of lipid	1,96E-03	*ACLY*, *CAT*, *FASN*, *HMGCR*, *LDLR*
Quantity of monounsaturated fatty acids	2,14E-03	*ACLY*, *ELOVL5*
Esterification of lipid	2,42E-03	*EIF4EBP1*, *FABP3*, *LDLR*
Abnormal quantity of triacylglycerol	2,56E-03	*CD36*, *LDLR*
Concentration of malonyl-coenzyme A	2,56E-03	*ACACB*, *ACLY*
Synthesis of acetyl-coenzyme A	2,56E-03	*ACLY*, *FASN*
Transport of palmitic acid	2,56E-03	*CD36*, *FABP3*
Oxidation of fatty acid	2,81E-03	*ACACB*, *CD36*, *FABP3*, *HADHA*, *HIF1A*
Regulation of steroid	4,02E-03	*LDLR*, *PLAT*
Metabolism of membrane lipid derivative	4,27E-03	*CAT*, *EEF1A1*, *FABP3*, *FASN*, *GNPAT*, *HMGCR*, *LDLR*, *RAC1*
Concentration of corticosterone	4,73E-03	*IGF2*, *KCNMA1*, *LDLR*, *PLAT*
Uptake of palmitic acid	5,17E-03	*CD36*, *FABP3*
synthesis of long chain fatty acid	5,79E-03	*CAV2*, *FASN*
Synthesis of phospholipid	6,34E-03	*EEF1A1*, *FABP3*, *FASN*, *GNPAT*, *RAC1*
Accumulation of C10:1 dicarboxylic acid	6,40E-03	*HADHA*
Accumulation of myristic acid	6,40E-03	*HADHA*
Accumulation of C14:1 fatty acid	6,40E-03	*HADHA*
Accumulation of sebacic acid	6,40E-03	*HADHA*
Accumulation of suberic acid	6,40E-03	*HADHA*
Activation of 5, 6-dichloro-7,7,7-trifluoro-4-thia-5-heptenoyl-coenzyme A	6,40E-03	*HADHA*
Conversion of 3-hydroxy-3-methylglutaryl-coenzyme A	6,40E-03	*HMGCR*
Conversion of palmitic acid	6,40E-03	*FASN*
Cross-linkage of phosphatidylserine	6,40E-03	*ANXA5*

Biological functions were determined using Gene Ontology

### Validation by real-time RT-PCR of a subset of genes revealed by differential microarray analysis

The Table 5 summarises the gene expression results derived for selected genes encoding lipogenesis-related. The microarray screening indicated that restricted feeding strategy down-regulated the expression of *ACACB* (FC = 1.32), *FABP3* (FC = 1.45), *HADHA* (FC = 1.41) and *SLC37A4* (FC = 1.40) genes, when compared to the CG system (FDR<0.05). In contrast, in the CG bulls the expression of *ELOVL5* (FC = 1.58) and *FASN* (FC = 1.71) was down-regulated when compared to DG bulls (FDR<0.05). These results were confirmed to be significant in the case of *ELOVL5* (*P* = 0.024), *FASN* (*P* = 0.030) and *SLC37A4* (*P* = 0.027), and approached significance in the case of *FABP3* (*P* = 0.067) by qRT-PCR analysis. Similar trends and magnitudes of change were observed for *ACACB* and *HADHA* by both methodologies, although significant for microarray only. *SCD1* and *SCD5* gene expressions were not found to be affected by growth path, through microarray or RT-qPCR gene expression analysis (*P*>0.05).

**Table 5:** Up and downregulated genes: Fold change for longissimus thoracis muscle mRNA detected in response to continuous (CG, n = 16) or discontinuous (DG, n = 16) growth production system.

Symbol	Microarray Fold Change (DG/CG)	*P* value	FDR	RT-qPCR Fold Change (DG/CG)	*P* value
*ACACB*	↓1.32	0.010	0.047	↓1.52	0.275
*ELOVL5*	↑1.58	0.002	0.034	↑1.61	0.024
*FABP3*	↓1.45	0.002	0.041	↓1.33	0.067
*FASN*	↑1.71	0.001	0.039	↑1.48	0.030
*HADHA*	↓1.41	0.002	0.038	↓1.11	0.681
*SCD1*	↓1.26	0.158	0.341	↓1.05	0.813
*SCD5*	↓1.14	0.391	0.570	↓1.23	0.834

## Discussion

### Muscle lipid content and fatty acid composition

Several studies were focused on the biological mechanisms and effects of compensatory growth on energy partition and tissue growth [1, 6, 10, 15]. Compensatory growth is characterized, after a feed restriction period, by greater growth rates than control animals of the same age. This phenomenon is associated to an accelerated turnover and increase in protein synthesis during refeeding. The higher nutrient supply improves the functionality of the somatotropic axis, increasing the concentration of anabolic hormones and a rapid muscle deposition [23]. The post-restriction period has been reported to be associated with greater expression of genes involved in cellular function and organization, contributing to an increased build up capacity within the tissue for subsequent accelerated growth and protein accretion [10]. In addition, fat metabolism is also regulated thereby affecting meat nutritional and sensory attributes. In ruminants the PUFA are preferentially esterified into structural phospholipids whereas saturated and monounsaturated fatty acids including the *trans*-biohydrogenation intermediates (*trans*-BI) are mainly deposited in triacylglycerols [24, 32]. Hence, leaner meat has greater PUFA and lesser *trans*-BI proportions than fatty meat. In the present experiment, both groups were fed the same finishing diet in the last 9 months prior to slaughter and previous dietary effects, evaluated at slaughter, are expected to have been diluted or no longer exist [10]. The intramuscular fat content in Lt muscle was similar between DG and CG animals. Thus, the proportion between phospholipids and triacylglycerols can be expected to be identical between experimental groups in accordance to previous results [7, 8, 25, 26]. However, the response on metabolic activity and intramuscular lipid content to re-feeding after a restriction period depended on the studied muscle [6]. The feed restriction followed by compensatory growth in Lt muscle from Alentejana bulls was a source of variation in IMF fatty acid composition, suggesting that lipid metabolism was affected by growth path. These changes in muscle fatty acid composition depend on rate of fatty acid deposition and mobilization. During the restriction period the expression of genes associated with the beta oxidation of fatty acids are significantly increased, when compared to non-restricted animals, due to a greater energy production from lipid stores [10]. During re-feeding gene expression related to expression of fatty acid synthesis predominates over degradation. In a study from Keogh et al. [10], there was a down-regulation of *CPT1B* gene during re-feeding, which encodes the rate-controlling enzyme of beta oxidation in muscle mitochondria, suggesting a greater propensity for triacylglycerols synthesis. Such capability involved the expression of genes that encodes for the Acetyl-CoA carboxylase (ACC), which catalyzes the carboxylation of acetyl-CoA to malonyl-CoA, the rate-limiting step in fatty acid synthesis, and the enzymes involved in fatty acid elongation (ELOVL). This was confirmed in the present study. It was observed an enhanced activity of lipogenic enzymes in the DG bulls, when compared to the CG counterparts. The ACC, is regulated by phosphorylation and dephosphorylation. This is a control mechanism, which together with cellular metabolites such as CoA, citrate, and palmitoyl-CoA serves to fine-tune the synthesis of long-chain fatty acids under different physiological conditions [56]. The acetyl-CoA carboxylase beta (*ACACB*) and ELOVL fatty acid elongase 5 (*ELOVL5*), as well as *FASN* and ATP citrate lyase (*ACLY*), which have a key role in FA metabolism [27–29], were differentially expressed between experimental groups (Table 3). In contrast to Keogh et al. [10], no differences in the expression of *DGAT*, *ELOVL6* and *SLC27A6* genes, that encodes enzymes envolved in FA synthesis, were observed between CG and DG bulls. The longer (9 months) re-feeding period that was implemented here, in comparison to Keogh et al. [10], may have masked the effects of compensatory growth. Some of the genes that may have been up-regulated in the outset of the compensation may no longer have an important role at the time of slaughter.

Nonetheless, changes in muscle FA composition cannot be exclusively attributed to enzyme activity. Ruminant meat fatty acid composition is complex and affected by multiple factors including the diet`s composition, rumen microbial metabolism, besides genetic factors [30,31,33]. In addition to *de novo* synthesis in the adipocytes, part of FA derives from rumen lipid metabolism and include the odd- and branched-chain fatty acids that are synthetized *de novo* by ruminal bacteria. Furthermore, ruminant fat depots also include *trans*-BI which are intermediate compounds from microbial biohydrogenation pathways [31]. Changes in the deposition of these fatty acids may reflect rumen microbial population shifts. Muscle from DG had less branched chain FA of rumen microbial origin and more *trans* 18:1 isomers (Table 2), particularly 18:1 *trans*-10 (18:1*t*10) than CG bulls. In general, the main *trans*-BI generated during rumen biohydrogenation is the 18:1 *trans*-11, but when ruminants are fed low-fiber and high starch diets a shift on biohydrogenation pathways is often observed where 18:1 trans-11 is replaced by 18:1 *trans*-10 as the major *trans*-BI [57, 58]. These change in the rumen biohydrogenation pathways, known as *trans*-10 shift, and has been clearly associated with milk fat depression in dairy cows [57,59]. The increased accumulation of trans-BI and the decreased 18:0 deposition in DG animals strongly suggest a more incomplete rumen biohydrogenation of dietary unsaturated FA in DG bulls. Furthermore, the greater predominance of 18:1 *trans*-10 and 18:1*t*10/18:1*t*11 ratio in the DG group, suggests a shift in the rumen biohydrogenation pathways (i.e. the *trans*-10 shift). The *trans*-10 shift is known to occur often in ruminants fed starch-rich diets, as cereal based concentrates, due to a modification in the ruminal microbial population structure [31]. The diet provided to both groups during the last 9 months before slaughter was rich in starch and could explain the high 18:1*t*10/18:1*t*11 ratio found in both experimental groups. However, the DG bulls exhibit large individual 18:1*t*10 and 18:1*t*10/18:1*t*11 ratios variability, suggesting instability in the rumen microbiota [33], probably related to differences in frequency of feed intake, feeding behavior or rumen acidosis episodes (not evaluated) between groups. It is well known that restricted animals undergo physiological changes, increasing their feed intake and enlarging digestive tract after *ad-libitum* access to feed, due to changes in feeding behavior [3]. Furthermore, besides ruminal size, the saliva production rate and rumen kinetics, as well as retention time could play a role in rumen microbial population composition [33]. These factors may affect animal productivity by changing the number and diversity of rumen microorganisms, the pattern of volatile FA, the composition and maintenance energy requirement of the microbes and can promote an establishment of rumen ecological equilibrium that favors the *trans*-10 shift.

In addition to rumen effects, the growth path was a noticeable source of variation of meat fatty acid composition due to its effects on delta-9 desaturation indices. When compared to CG animals, all the delta-9 desaturase ratios were greater in the DG group. However, these ratios are only a rough evaluation of the delta-9 desaturase activity, as most of the individual FA have at least a dual origin (i.e. dietary preformed FA and *de novo* synthesis). This is the case of the 18:0 fatty acid, which is reduced in DG animals because less is synthetised in the rumen (as discussed above) but probably also because more has been converted to 18:1*cis*-9.

### Differential expression genes

In a previous study, genes envolved in cytoskeleton and extracellular matrix were down-regulated in skeletal muscle after nutritional restriction [7followed by re-feeding, the main differential expression genes in Alentejana bulls were related to biological processes associated with lipid metabolism, nucleic acid metabolism, small molecule biochemistry, molecular transport and post-translational modifications (Table 3). However, protein expression vary according to animal type, muscle and breed [34, 35]. Similarly, gene expression differs significantly between longissimus muscles from different beef cattle breeds [36]. In addition, gene expression is also influenced by the diet composition [37]. Hence, even if animal performances reported in this study are similar to those previously reported by Cuvelier et al. (2006) [38] for young bulls from other beef breeds, the results of this study cannot be extrapolated to other livestock management systems.

Among genes with a fundamental role in lipid metabolism, the ACACB gene is the major form expressed in heart and skeletal muscles [40] and encodes the complex multifunctional enzyme system acetyl-CoA carboxylase, which is involved in the regulation of both FA oxidation and biosynthesis, catalysing the carboxylation of acetyl-CoA to malonyl-CoA. This enzyme is thought to control FA oxidation by means of the ability of malonyl-CoA to inhibit carnitine-palmitoyl-CoA transferase I, the rate-limiting step in FA uptake and oxidation by mitochondria [39,40]. The ACACB knock-out mice was reported to exhibited a high FA oxidation rate and a reduced fat content [40]. In agreement with the findings of Keogh et al. [10], when compared to CG bulls, the DG bulls had lower expression levels of ACACB, which could help to explain the lower contents of 18:0 and SFA in DG bulls.

As regards fatty acid synthesis, when compared to the CG bulls, both FASN and ELOVL5 genes were down-regulated in the CG. The FASN encodes a multifunctional protein whose main function is to catalyse the synthesis of palmitate from acetyl-CoA and malonyl-CoA and synthesises saturated FA up to 16 carbons in length [41]. These endogenous FA, as well as the diet´s FA could be further elongated into long chain FA containing 18 or more carbon atoms by the ELOVL5 [42]. Among other enzymes and proteins, the FASN expression has been directly associated with marbling [29]. However, in contrast to these authors, FASN was differentially expressed between CG and DG Alentejana bulls without differences in IMF content probably due to the involvement of other key enzymes. The elongation of very long chain FA genes (ELOVL) encodes for enzymes that play an important role in elongation of long-chain fatty acids [42, 43]. The ELOVL5 catalyses the first and rate-limiting reaction of the long-chain FA elongation cycle, elongating C18 and C20 PUFA that are involved in multiple biological processes as precursors of membrane lipids and lipid mediators, with low activity towards C22 [42,43]. When compared to CG bulls, the increased ELOVL5 gene expression observed in DG group, may explain, at least in part, the greater 22:4n-6 and 22:6n-3 PUFA percentages. However, no differences between CG and DG bulls were observed in total PUFA, regardless of the higher FABP3 expression levels in the CG group. Among others, the intracellular FA-binding proteins (FABPs) are thought to participate in the uptake, intracellular metabolism and/or transport of long-chain fatty acids [44]. They may also contribute to the modulation of cell growth and proliferation. During early differentiation in bovine-derived adipocytes, the *FABP3* gene is usually up-regulated [45]. However, the function of *FABP3* is not entirely known. *In vitro* studies using hepatocytes found that rates of FA uptake and metabolism were increased when *FABP* levels were higher [46].

The *HADHA* gene encodes the alpha subunit of the mitochondrial trifunctional protein, which catalyses the last three steps of mitochondrial beta-oxidation of long chain FA [47]. This mitochondrial tri-functional protein is required to metabolize the long-chain FA, which are also an important energy source for the liver and other tissues during periods of fasting. The lower HADHA mRNA levels in the DG is concomitant with decreased long-chain FA metabolism in the latter. It is possible that bulls undergone a compensatory growth phase could spare long chain FA such as 22:0 and the essential fatty acids: 22:4n-6 and 22:6n-3.

The stearoyl-CoA desaturase (SCD) is a rate-limiting enzyme responsible for the conversion of SFA into monounsaturated fatty acids (MUFA).[48]. The SCD is also a key enzyme in the endogenous production of the *cis*-9, *trans*-11 isomer of conjugated linoleic acid (CLA) [49]. To date, two isoforms of stearoyl-CoA desaturase have been identified in bovine, SCD1 and SCD5, both expressed in bovines [50]. When compared to CG bulls, the greater desaturation indices for stearoyl-CoA desaturase in DG bulls suggests that growth path was a significant source of variation on SCD1 and SCD5 mRNA levels. However, we did not observe any significant differences in the expression of both genes. The discrepancy between gene expression levels and the desaturation indices could be due to post-transcriptional regulatory processes that lead to differences in the amount of protein produced or to inhibition of enzyme activity [51]. Furthermore, the desaturation indices for stearoyl-CoA desaturase reflect a fatty acid accumulation over a period of several months whilst the mRNA levels were evaluated at slaughter. It is possible that *SCD1* and *SCD5* mRNA levels have been greater in DG, when compared to CG bulls, in the last months before slaughter. Furthermore, growth path was a source of variation of *SLC37A4* mRNA levels which were lower in DG bulls, suggesting an effect on glucose metabolism that could help to explain the differences found between CG and DG bulls in desaturase activity. The *SLC37A4* gene regulates glucose-6-phosphate transport from the cytoplasm to endoplasmic reticulum lumen, in order to maintain glucose homeostasis [52]. It has been reported an association between glucose metabolism and desaturation activity [53], reinforcing the possibility that *SCD1* and *SCD5* mRNA levels were increased in DG bulls before slaughter. Furthermore, *FASN* as well as *SCD* are strong candidate genes influencing FA composition in beef cattle [28, 29].

## Conclusions

Results from the present study showed that growth path influences fatty acid metabolism with repercussions on *longissimus thoracis* muscle IMF composition, with beneficial effects on meat nutritional quality. When compared to continuous growth bulls, meat from the discontinuous group had greater levels of 18:1 isomers and lower levels of branched chain fatty acids, which could reveal an important impact of discontinuous growth on rumen microbial metabolism. Increased expression of genes associated with fatty acid beta-oxidation where shown during feed restrictionwhereasduring re-feeding predominate the expression of genes associated with fatty acid synthesis. This pattern can be observed in Alentejana bulls, after 9 months of compensatory growth,. When compared to continuous growth bulls some genes such as ELOVL5 and FASN, which have a key role in fatty acid syntheses and elongation, were up-regulated in discontinuous growth bulls. It was observed a clear trend on delta-9 desaturase activity indices, which were consistently higher in discontinuous bulls, suggesting an important effect of growth path on *SCD1* and *SCD5* mRNA levels. However, this was not associated to differences in the expression of both genes. As for others, it is possible that these genes may have an important role in fatty acid metabolism during refeeding but are no longer up-regulated at slaughter. These results contribute to the knowledge about the mechanisms involved in fatty acid metabolism during compensatory growth at a molecular level and could help to identify genomic biomarkers that can be used in the future in the selection of cattle used in traditional beef production systems in Mediterranean areas.

## Supporting information

S1 Table Normalized data. (XLS)

S2 Table Gene expression analysis. (XLS)
