# Correction: Does growth path influence beef lipid deposition and fatty acid composition?

**DOI:** 10.1371/journal.pone.0201997

**Published:** 2018-08-02

**Authors:** Ana S. H. Costa, Paulo Costa, Susana P. Alves, Cristina M. Alfaia, José A. M. Prates, Veronica Vleck, Isabelle Cassar-Malek, Jean-François Hocquette, Rui J. B. Bessa

In the Funding section, the individual fellowship number to A. S. C. is listed incorrectly. The correct fellowship number is: SFRH/BD/61068/2009.

There is an error in first sentence of the “Differential expression genes” section of the Results. The correct sentence is: “In a previous study, genes involved in cytoskeleton and extracellular matrix were down-regulated in skeletal muscle after nutritional restriction [7]. After a period of feed restriction followed by re-feeding, the main differential expression genes in Alentejana bulls were related to biological processes associated with lipid metabolism, nucleic acid metabolism, small molecule biochemistry, molecular transport and post-translational modifications (Table 3).”

There is an error in reference 1. The correct reference is: Hornick JL, Van Eenaeme C, Clinquart A, Diez M, Istasse L. Different periods of feed restriction before compensatory growth in Belgian Blue bulls: I. Animal performance, nitrogen balance meat characteristics, and fat composition. Journal of Animal Science. 1998; 76:249–59. pmid:9464906

In Table 2, the fourth column is mistakenly included. Please see the correct Table 2 here.

**Table 2:** Intramuscular fat (IMF, g/100g meat), total fatty acids (total FA, g/100g muscle) and fatty acid composition (% of total FA) of muscle in continuous growth (CG) and discontinuous growth (DG).

	Group		*P-value*
	CG (n = 20)	DG (n = 16)	
IMF	1.87±0.15	1.93±0.17	0.807
Total FA	1.46±0.12	1.60±0.13	0.437
*Fatty acids*			
14:0	2.15±0.11	2.06±0.10	0.608
14:1c9	0.32±0.03	0.36±0.03	0.404
*i*-15:0	0.09±0.005	0.06±0.004	<0.001
*a*-15:0	0.15±0.006	0.12±0.005	0.009
15:0	0.29±0.01	0.32±0.01	0.183
*i*-16:0	0.13±0.007	0.11±0.007	0.017
16:0	24.1±0.4	23.6±0.3	0.362
16:1c7	0.17±0.003	0.18±0.006	0.237
16:1c9	2.57±0.13	2.76±0.15	0.376
*i*-17:0	0.35±0.03	0.31±0.02	0.200
*a*-17:0	0.51±0.03	0.43±0.02	0.033
17:0	0.96±0.03	1.01±0.03	0.266
17:1c9	0.60±0.02	0.79±0.03	<0.001
*i*-18:0	0.10±0.004	0.09±0.003	0.187
18:0	17.9±0.4	15.3±0.4	<0.001
18:1t6+t8	0.16±0.01	0.19±0.01	0.052
18:1t9	0.20±0.01	0.26±0.02	0.010
18:1t10	0.52±0.07	1.91±0.34	<0.001
18:1t11	0.53±0.03	0.40±0.02	0.003
18:1c9	29.6±0.8	29.3±0.8	0.731
18:1c11	1.68±0.06	1.93±0.07	0.003
18:1c12	0.33±0.04	0.30±0.04	0.658
18:1c13	0.14±0.009	0.19±0.01	0.004
18:1t16+c14	0.13±0.004	0.09±0.006	<0.001
18:1c15	0.04±0.04	0.05±0.04	0.087
18:2n-6	9.99±0.59	9.95±0.55	0.967
18:3n-3	0.43±0.02	0.41±0.02	0.471
18:3n-6	0.05±0.005	0.08±0.006	<0.001
20:0	0.11±0.003	0.09±0.002	0.005
20:1c11	0.04±0.002	0.04±0.002	0.984
CLA(c9t11)	0.09±0.008	0.10±0.009	0.722
20:2n-6	0.08±0.004	0.08±0.004	0.169
20:3n-9	0.11±0.01	0.11±0.009	0.566
20:3n-3	0.02±0.001	0.03±0.002	0.157
20:4n-6	2.75±0.19	3.35±0.25	0.057
20:5n-3	0.13±0.02	0.14±0.02	0.491
22:0	0.48±0.03	0.66±0.05	0.003
22:4n-6	0.31±0.02	0.38±0.03	0.029
22:5n-3	0.35±0.03	0.41±0.03	0.198
22:6n-3	0.03±0.004	0.05±0.004	0.026
*Partial sums*			
SFA	46.0±0.7	43.1±0.6	0.002
*cis*MUFA	35.5±0.8	35.9±0.8	0.765
TFA	1.64±0.10	2.95±0.33	0.001
BCFA	1.34±0.07	1.13±0.05	0.013
PUFA	14.2±0.8	15.0±0.8	0.529
n-3	0.53±0.05	0.62±0.05	0.199
n-6	13.2±0.8	13.9±0.8	0.550
*Desaturation indices*			
ID14	12.7±0.7	14.6±0.8	0.086
ID16	9.39±0.30	10.7±0.38	0.016
ID17	38.1±0.1	44.3±0.2	<0.001
ID18	61.6±0.7	66.0±0.9	<0.001
IDCLA	15.1±1.1	20.1±1.6	0.017

IMF = intramuscular fat; FA = fatty acids; SFA = saturated fatty acids (sum of 14:0, 15:0, 16:0, 17:0, 18:0 and 20:0); cisMUFA = monounsaturated fatty acids (sum of 14:1c9, 16:1c7, 16:1c9, 17:1c9, 18:1c9, 18:1c11, 18:1c12, 18:1c13, 18:1c15, 19:1 and 20:1c11); TFA = trans fatty acids (sum of 18:1t6-t8, 18:1t9, 18:1t10, 18:1t11, 18:1t12, 18:1t16, c14 and 18:2t11c15); BCFA = branched chain fatty acids (sum of i-14:0, i-15:0, a-15:0, i-16:0, i-17:0, a-17:0 and i-18:0 (*a*- = anteiso *i*- = iso)); PUFA = polyunsaturated fatty acids (sum of 18:2n-6, 18:3n-6, 18:3n-3, CLA, 20:3n-3, 20:5n-3, 22:5n-3, 22:6n-3; 20:2n-6, 20:4n-6 and 22:4n-6); n-3 = sum of 18:3n-3, 20:3n-3, 20:5n-3, 22:5n-3 and 22:6n-3; n-6 = sum of 18:2n-6, 18:3n-6, 20:2n-6, 20:4n-6 and 22:4n-6; ID14:0 = (14:1c9×100)/(14:0+14:1c9); ID16:0 = (16:1c9×100)/(16:0+16:1c9); ID18:0 = (18:1c9×100)/(18:0+18:1c9); IDCLA = (18:2c9,t11×100)/(18:1t11+18:2c9,t11). Means in the same row with different superscripts are statistically different (P<0.05).
